# Global Consensus From Clinicians Regarding Low Back Pain Outcome Indicators for Older Adults: Pairwise Wiki Survey Using Crowdsourcing

**DOI:** 10.2196/11127

**Published:** 2019-01-15

**Authors:** Arnold YL Wong, Henrik H Lauridsen, Dino Samartzis, Luciana Macedo, Paulo H Ferreira, Manuela L Ferreira

**Affiliations:** 1 Department of Rehabilitation Sciences Hong Kong Polytechnic University Hong Kong China (Hong Kong); 2 Department of Sports Science and Clinical Biomechanics University of Southern Denmark Odense Denmark; 3 Rush University Medical Center Chicago, IL United States; 4 Population Health Research Institute Hamilton, ON Canada; 5 Department of Physiotherapy, University of Sydney Sydney Australia; 6 Institute of Bone and Joint Research University of Sydney Sydney Australia

**Keywords:** crowdsourcing, wiki survey, low back pain, older people, outcome indicators

## Abstract

**Background:**

Low back pain (LBP) is one of the most debilitating conditions among older adults. Unfortunately, existing LBP outcome questionnaires are not adapted for specific circumstances related to old age, which may make these measures less than ideal for evaluating LBP in older adults.

**Objective:**

To explore the necessity of developing age-specific outcome measures, crowdsourcing was conducted to solicit opinions from clinicians globally.

**Methods:**

Clinicians around the world voted and/or prioritized various LBP outcome indicators for older adults on a pairwise wiki survey website. Seven seed outcome indicators were posted for voting while respondents were encouraged to suggest new indicators for others to vote/prioritize. The website was promoted on the social media of various health care professional organizations. An established algorithm calculated the mean scores of all ideas. A score >50 points means that the idea has >50% probability of beating another randomly presented indicator.

**Results:**

Within 42 days, 128 respondents from 6 continents cast 2466 votes and proposed 14 ideas. Indicators pertinent to improvements of physical functioning and age-related social functioning scored >50 while self-perceived reduction of LBP scored 32.

**Conclusions:**

This is the first crowdsourcing study to address LBP outcome indicators for older adults. The study noted that age-specific outcome indicators should be integrated into future LBP outcome measures for older adults. Future research should solicit opinions from older patients with LBP to develop age-specific back pain outcome measures that suit clinicians and patients alike.

## Introduction

Low back pain (LBP) is a debilitating condition [1,2] that causes functional decline in older adults [3]. The predicted percentage of adults aged 60 years and over will triple by 2050 [4], which may inevitably increase incidences of noncommunicable conditions (including musculoskeletal disorders) [5]. It has been estimated that 30% of seniors aged 65 years and over in the United States live with LBP [6].

Since the sequelae of LBP has larger impacts on physical function and quality of life of older adults than younger individuals [7,8], it is essential to effectively treat the affected older adults. Unfortunately, the efficacy of different LBP interventions in older adults remains uncertain because many clinical trials on LBP interventions exclude older patients [9], and existing LBP outcome measures do not consider age-related physical and psychosocial changes in older adults and may not comprehensively evaluate the impact of LBP on those older adults [3,10]. Although more studies have evaluated the efficacy of various LBP interventions on older adults [11,12], there is no consensus regarding the necessity of developing age-specific outcome measures for older adults with LBP. Some clinicians believe that LBP outcome indicators for older adults should not differ from those for young adults, whereas others argue that older adults need another set of LBP outcome indicators given their comorbidities and altered psychosocial conditions [13,14]. Given the controversy, it is important to broadly solicit clinicians’ opinions on the importance of various key LBP outcome indicators to determine the necessity of developing new or adapting existing LBP outcome measures for older adults.

Crowdsourcing is a research approach collating information and solutions from a group of people or experts using the internet in a controlled manner. Specifically, an organization presents a complex problem to a specific group of internet users who will provide solutions to the challenge or problem on a voluntary or employee-paid basis. The organizer then analyzes the findings for further applications [15]. Crowdsourced results are highly applicable to the target audience and end users because they are involved in deriving the solutions [15]. Multiple health disciplines have adopted crowdsourcing to monitor disease outbreaks, analyze gene expression data, interpret medical images, or record drug responses [16-18]. Collectively, crowdsourcing can facilitate knowledge translation and inform biomedical research [19].

Our study aimed to use a crowdsourcing approach to identify global clinicians’ opinions regarding the relative importance of various LBP outcome questionnaire indicators for older adults.

## Methods

### Creation of a Pairwise Wiki Survey

Our study adopted a pairwise wiki survey approach via a crowdsourcing method, which allows prioritization of ideas [20]. Briefly, a pairwise wiki survey involves a single question with multiple potential answers. Respondents contribute to the survey by (1) making pairwise comparisons between two randomly presented answers (ie, voting between two ideas) and/or (2) adding new ideas for future respondents to vote. This approach quantifies responses based on the relative priority of different answers from all respondents and integrates respondents’ new ideas for prioritization (vote up or down) using an established algorithm [20]. Unlike traditional surveys, respondents do not confine their responses to the choices offered by the researchers [21]. Therefore, influences of researchers’ preexisting knowledge or biases are minimized during data collection [20].

**Figure 1:**
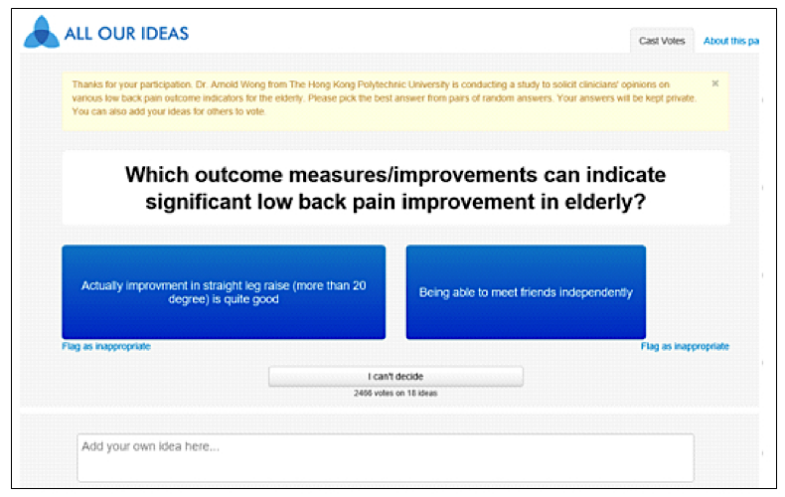
Research objective and research question.

A pairwise wiki survey was created on a free open-source website, All Our Ideas (www.allourideas.org), to let respondents vote on ideas about “Which outcome measures/improvements can indicate significant low back pain improvement in elderly?” [22]. A brief description of the research objective along with the research question (Figure 1) and 7 seed answer items were posted on the website for voting on June 12, 2016. The 7 seed answers were determined by a panel of clinicians with 7 to 22 years of relevant clinical experience and are as follows:

- Able to walk independently with or without walking aids
- Able to do grocery shopping without significant increase in pain
- No longer requires support from caregivers
- Able to take care of grandkids
- Able to meet friends independently
- Doesn’t need to see physicians/clinicians because of low back pain
- At least a 2-point decrease in pain on visual analogue scale

The panel comprised a physiotherapist specializing in spinal pain management, a physiotherapist specializing in geriatric rehabilitation, an orthopedic surgeon, and a geriatrician. These seed answers were aligned with the core set of outcome domains (physical functioning, pain intensity, and health-related quality of life) derived from a Delphi study for measuring and reporting nonspecific LBP in clinical trials [23]. To evaluate the relevance of age-related outcome indicators in assessing LBP improvements of older adults, an age-specific outcome indicator (ie, being able to take care of grandkids) was added as one of the 7 seed answers. Only 7 clinically relevant seed answers were included because they were used as catalysts to stimulate constructive contributions from respondents and minimize biases from the panel. Respondents were encouraged to contribute their new ideas about potential LBP outcome indicators for older adults on the website (Figure 1). The primary investigator determined the appropriateness of the ideas submitted by respondents. Respondent-contributed answers were deactivated for voting if they were duplicates of existing answers/ideas, irrelevant to the question of interest (ie, LBP outcome indicators for older adults), or comments/questions about the appropriateness of the study design, website, answers, or research objectives. This study was approved by an ethics board committee and conducted according to the Declaration of Helsinki.

To advertise the survey to targeted clinicians (ie, physiotherapists, occupational therapists, chiropractors, osteopaths, physicians, nurses, physicians, gerontologists, and general practitioners), 3 strategies were adopted. First, standardized messages with the survey hyperlink were posted on the Facebook accounts of multiple health care professional organizations (Multimedia Appendix 1) identified using various key words: chiropractic, chiropractors, general practice, general practitioners, geriatric, geriatricians, gerontological, gerontology, manual therapists, manual therapy, medical, medicine, nurses, nursing, orthopaedics, orthopedics, physical therapists, physical therapy, physiotherapists, physiotherapy, osteopathic, osteopathy, occupational therapists, or occupational therapy. Briefly, the Facebook message explained that a group of researchers was conducting a survey to solicit clinicians’ opinions regarding various LBP outcome indicators for older adults with LBP and that respondents could contribute to the online survey by selecting their preferred outcome indicators or suggesting new outcome indicators (Textbox 1). Second, similar key words were used to identify various target groups (Multimedia Appendix 1) and a standardized Tweet message alongside the hashtag of these groups was used to advertise the survey (Textbox 1). A second round of the advertisement was sent to the same social media sites on July 3, 2016. Third, the primary investigator sent personal messages through Facebook messenger to invite 15 lead clinicians and clinician-scientists (orthopedic specialists, physiotherapists, chiropractors, and nurses) in Australia, Canada, Hong Kong, Denmark, Norway, and the United States to cast their votes and share the survey hyperlink on their personal Facebook pages or the Facebook pages of their respective local professional organizations. Only a small number of personal messages were sent because this pilot study mainly aimed to use social media to promote the survey.

**Standardized Facebook and Twitter messages that were posted or linked to various physiotherapy, chiropractic, osteopathic, occupational therapy, medical, and gerontology professional groups.**

Facebook

- A group of researchers is conducting a crowdsourcing research project to understand clinicians’ opinions regarding the key outcome indicators that represent low back pain improvements in older adults aged 65 years and over. The results can help develop tailored outcome measures for older adults. If you are willing to help, please click on the link and cast your votes. Your participation is voluntary. When you click on the link, you will see two potential answers that indicate significant improvements of low back pain in older adults. You are requested to pick the best answer from the two options. Once you submit your answer, another two random outcome options will be shown for comparison. The procedure will be repeated until you quit. Your answers will be kept strictly confidential. You can also add new ideas of outcome indicators for others to vote. Please feel free to share the link with your colleagues. Thanks in advance for your help. allourideas.org/olderpeoplewithlowbackpain

Twitter

- Please cast your vote to help develop new low back pain outcome measures for the elderly allourideas.org/olderpeoplewithlowbackpain

**Figure 2:**
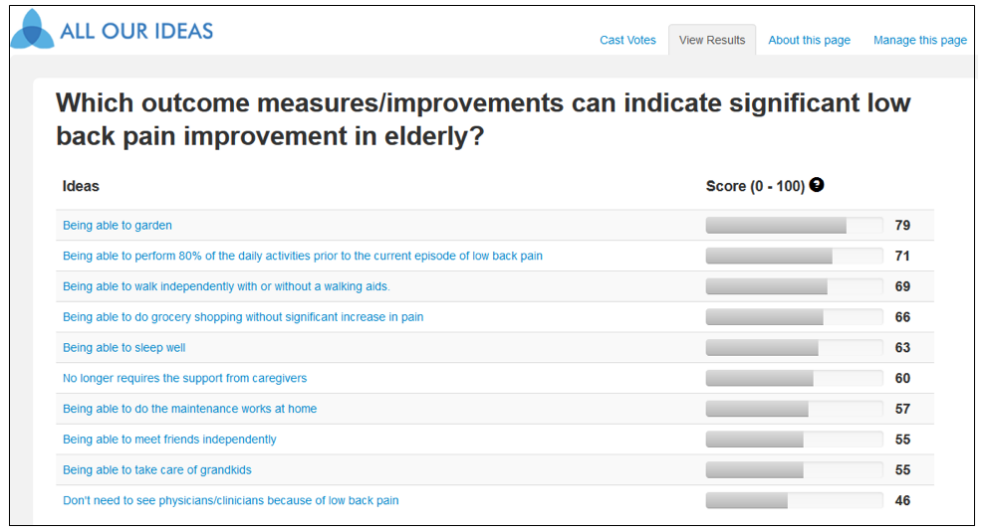
Resulting scores of all the answer items displayed on the website.

### Data Analysis

The website uses a published algorithm to estimate the chance of a given answer item in beating another randomly presented item for a randomly chosen respondent [20]. Briefly, a binomial model was chosen to estimate the probability of a win for each answer item. Assuming a uniform prior probability for a binomial variable, the resulting posterior probability to a win follows a beta distribution [24]. By multiplying the expected value of that beta distribution by 100, the resulting estimated score (ranging from 0 to 100) would represent the winning percentage of a given item. If a given item scores 0, it is expected to lose for all pairwise comparisons. Conversely, if an item scores 100, it is anticipated to always win. The resulting scores of all the answer items are displayed on the website (Figure 2).

Additionally, raw data (ie, the number of responses of each respondent, actual responses of each respondent, time spent on each comparison, number of new ideas from each participant, and response time of each respondent) were downloaded from the website for descriptive analysis using SPSS Statistics version 20.0 (IBM Corp). The binomial confidence interval of the mean score of each answer was also calculated [25].

## Results

### Number of Respondents and Responses

Over 42 days, 128 respondents contributed 2466 responses. During the same period, 179 visitors visited the website without casting any vote (a response rate of 41.7%). Respondents came from 60 cities in 22 countries on 6 continents (Table 1). The United States, China (Hong Kong), Australia, Canada, and Great Britain were the top 5 countries with the highest number of responses (range 239-541) and respondents (range 10-31).

**Figure 3:**
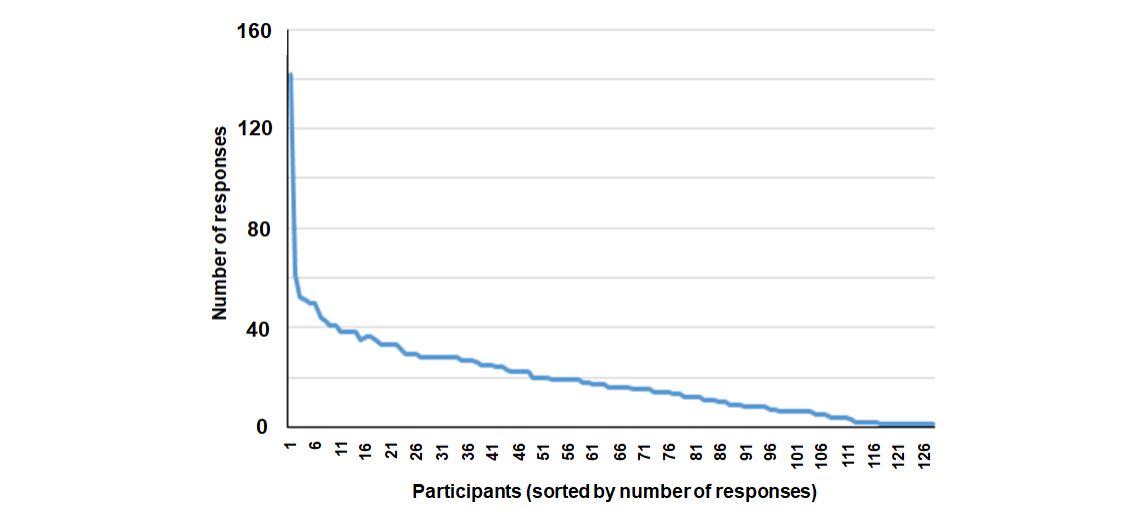
Distribution of responses per participant.

The median number of responses per respondent was 17 (range 1-142) (Figure 3). The median time spent on each comparison by the respondent was 4.7 seconds (range 0.4-314.9 seconds). Fourteen new ideas were proposed by the respondents (Table 2). Six respondents suggested 1 new idea each, 1 proposed 2, and 1 proposed 6. Nine out of 14 new ideas were proposed within the first 3 days of the survey, but the last active idea (If trunk flexion is indicated as a significant factor increasing low back pain in the first assessment, then straight leg raise would be one of the indicators) was proposed on the day 35. Three contributed ideas were not activated for voting because they were deemed to be inappropriate or duplicate. Given the respondent-contributed ideas, the number of active ideas in the survey increased from 7 to 18. Sixteen activated ideas were self-reported outcome indicators and 2 were related to physical examinations. The median number of times each activated idea was presented to respondents for comparison was 585 (range 55-686).

### Prioritization of Answers

**Figure 4:**
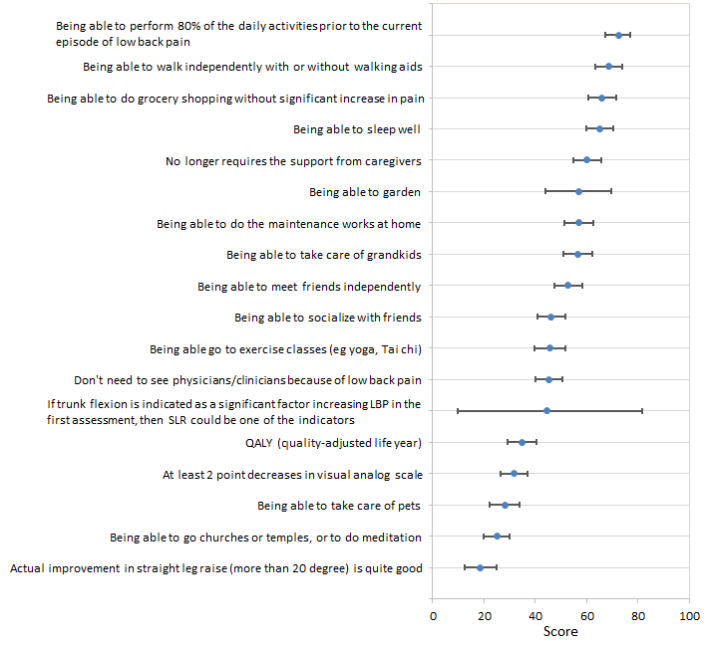
Rank scores of various potential low back pain outcome indicators for geriatric patients as estimated by the established algorithm on the website. LBP: low back pain; SLR: straight leg raise.

Nine out of 18 activated answer items scored more than 50, implying that these answers had a more than 50% chance of beating other answers in pairwise comparisons. The top 3 high-scoring ideas (able to perform 80% of the daily activities prior to the current episode of LBP, able to walk independently with or without walking aids, and able to do grocery shopping without significant increase in pain) had mean scores of 72, 69, and 66, respectively. Two of the top 5 high-scoring ideas were suggested by respondents (Figure 4). As hypothesized, outcome indicators related to pain and physical impairments yield only low scores. Specifically, the items “at least a 2-point decrease in visual analogue scale” and “actually improvement in straight leg raise (more than 20 degrees) is quite good” scored only 32 and 18, respectively.

**Table 1:** Number of responses and respondents by country.

Country	Responses (N)	Respondents (N)
United States	541	31
China and Hong Kong	433	21
Australia	420	19
Canada	320	15
Great Britain	239	10
Japan	98	4
Singapore	51	2
Netherlands	50	3
Rwanda	44	3
New Zealand	43	3
Brazil	37	2
Norway	34	2
Romania	31	2
Greece	28	2
Denmark	26	2
Colombia	25	1
Belgium	17	1
India	17	2
Switzerland	8	1
Trinidad and Tobago	3	1
Portugal	1	1

**Table 2:** Answer items created by the researchers and respondents/users.

Answer or idea items	Source	Status	Score
Able to walk independently with or without walking aids	Seed	Activated	69
Able to do grocery shopping without significant increase in pain	Seed	Activated	66
No longer requires support from caregivers	Seed	Activated	60
Able to take care of grandkids	Seed	Activated	57
Able to meet friends independently	Seed	Activated	53
Doesn’t need to see physicians/clinicians because of low back pain	Seed	Activated	44
At least a 2-point decrease in pain on visual analogue scale	Seed	Activated	32
Able to perform 80% of the daily activities prior to the current episode of low back pain	Respondent	Activated	72
Able to sleep well	Respondent	Activated	65
Able to garden	Respondent	Activated	57
Able to do maintenance work at home	Respondent	Activated	57
Able to socialize with friends	Respondent	Activated	46
Able to go to exercise classes (eg, yoga, tai chi)	Respondent	Activated	46
If trunk flexion is indicated as a significant factor increasing low back pain in the first assessment, then straight leg raise would be one of the indicators	Respondent	Activated	44
Quality-adjusted life year	Respondent	Activated	35
Able to take care of pets	Respondent	Activated	28
Able to go to church or temple or do meditation	Respondent	Activated	25
Actually improvement in straight leg raise (more than 20 degrees) is quite good	Respondent	Activated	18
I get the question but the semantics aren’t clear. Why should straight leg raise be an outcome measure for low back pain without mention of radiculopathy or sciatica?	Respondent	Deactivated	N/A^a^
Quality-adjusted life year	Respondent	Deactivated	N/A
The survey is overly repetitive. It will likely reduce your response rate. I have addressed the same issues more than 10 times	Respondent	Deactivated	N/A

^a^N/A: not applicable.

### Twitter Versus Facebook

Of 128 respondents referred to our wiki survey website, 94 (73.4%) were from Facebook and 34 (26.6%) were from Twitter. Most of the respondents (78/128, 60.7%) used a cell phone or computer tablet to participate in the survey; the rest (50/128, 39.3%) completed their surveys on their computers.

## Discussion

### Principal Findings

This is the first online crowdsourcing research to collect global clinician opinions regarding the relative importance of different LBP outcome indicators for older adults. As hypothesized, the majority of the respondents (clinicians) deemed that functional improvements were more important than improvements of pain or physical examinations. While some self-reported LBP outcome indicators identified in our study (eg, able to perform 80% of the daily activities prior to the current episode of LBP) might be true for other age groups, our respondents generally agreed that the age-specific functional outcome indicator (eg, able to take care of grandkids) was an important self-reported outcome indicator for older adults with LBP. These findings highlight that age-specific LBP outcome indicators, which have been ignored in existing self-reported LBP outcome measures, should be considered in the future development of new outcome measures for older adults with LBP.

Interestingly, 5 out of 7 seed answers derived from a panel of health care experts were deemed to be important by respondents. In fact, seed answers contribute to 56% of the answers scoring more than 50 points (Table 2). These results indicate that many clinicians around the world agreed on using certain seed answers to be the key LBP outcome indicators for older patients. Since several respondent-contributed outcome indicators were also rated as important, our study substantiates the feasibility and value of using a pairwise wiki survey to identify LBP outcome indicators for older people with LBP.

Participant responses were highly related to the advertising strategy. Since our study was mainly promoted on Facebook and Twitter accounts of various health care professional associations in the United States, Hong Kong, Australia, Canada, and Great Britain, greater response rates were attained from these regions. Interestingly, although our advertisements were posted on Facebook and Twitter accounts, 73.4% of the respondents were referred from Facebook, which indicates that Facebook was a more effective social media for recruiting clinician respondents in similar research than Twitter.

It is noteworthy that the confidence interval of one LBP outcome indicator (If trunk flexion is indicated as a significant factor increasing LBP in the first assessment, then straight leg raising test could be one of the indicators) was relatively large. This was attributed to the fact that this idea was received 7 days before the completion of data collection. Since this idea was only presented 55 times to respondents for comparison, its confidence interval was wide. Although this might affect the relative ranking of this outcome indicator, it would not affect the conclusion on the top priority outcome indicators because the most important outcome indicators should have been suggested at the early stage of the survey.

### Limitations

As with any clinical-based or survey type of research, inherent study limitations exist. Since the study did not involve older patients with LBP, our findings are limited to clinicians’ perspectives. Future research is warranted to solicit opinions from the target patient population during the process of developing a new LBP outcome measure for older adults.

Like many internet-based surveys, the study was limited by sample representativeness [26] because it did not collect participants’ detailed demographic information (eg, age, gender, years of education and clinical experience, health care disciplines). However, our respondents were highly likely to be clinicians because the survey was (1) not searchable on common search engines (eg, Google) unless the exact survey Web address was used, (2) only openly advertised on the Facebook and Twitter accounts of relevant professional bodies, and (3) promoted by personal emails sent to clinicians and clinician-scientists. This notion was further corroborated by the fact that the respondent-contributed ideas and voting results demonstrated high face validity to the research topic from the clinicians’ perspective.

The response rate of the study was 41.7%. In comparison, the response rate for Delphi studies that evaluated core outcome sets for LBP were between 45% and 52% [23]. This slight discrepancy might be attributed to the recruitment methods (open advertisements on social media vs personal invitations). Previous studies have found that response rates of internet surveys for clinicians are usually lower than traditional paper surveys [27-29]. While multiple reasons may explain the low response rate among clinicians (eg, lack of time, perceived low priority of surveys, and concerns about confidentiality) [29], response rates can be improved by sending multiple reminders or personalized letters [29,30]. Future studies should adopt multiple strategies (eg, incentives [31], personalized invitations [32], multiple reminders [30] and advertisements [33], or endorsements from professional associations [34]) to improve response rates and total number of respondents.

Since traditional surveys require researchers to determine all the details (eg, questions, orders of questions, and multiple plausible answers) prior to data collection, this top-down approach may introduce investigator biases and limit the knowledge that can be learned from respondents [20]. Conversely, our pairwise wiki survey used an ongoing collaborative approach to encourage respondents to create knowledge that was not anticipated by the researchers. Similar to a focus group that allows participants to react to others’ responses [35], user-contributed ideas collected from the wiki survey were continuously evaluated by future respondents. The success of this bottom-up interactive approach is reflected from our findings that respondents from all continents (except Antarctica) contributed 2 folds of new LBP outcome indicators within a short period of time and some of the proposed indicators were ranked as highly relevant LBP outcome indicators for older adults.

Our survey collected information based on the respondents’ eagerness to participate. While some respondents cast a single vote, others contributed heavily to the voting and/or new idea suggestions (Figure 3) [36]. Unlike traditional surveys that prohibit high contributors from answering extra questions and discard incomplete questionnaires from data analysis, a pairwise wiki survey collects as much or as little information as the respondent is willing to offer. Since wiki surveys value contributions from all respondents equally regardless of their time or effort spent on answering, wiki surveys may solicit more useful information from respondents than traditional surveys [20].

### Conclusions

Our study reveals a novel method for soliciting opinions from clinicians around the globe during the process of developing a new clinical outcome measure. Traditionally, the development of a new self-administered clinical outcome questionnaire involves a process of literature review, conduction of multiple focus groups or meetings among content experts (eg, clinicians, patients, scholars) to determine relevant items and/or domains in a questionnaire, and further modifications of items after pilot testing on target populations [37]. A pairwise wiki survey can be implemented as a low-cost adjunct survey tool to solicit ideas from a large population of clinicians or patients globally following the initial draft of items pooled from a panel of content experts. The survey results not only can broaden the perspectives to inform further panel discussions but allow rapid preliminary feedback from target users. However, further studies are warranted to evaluate the effectiveness of such an approach in improving the psychometric properties of the resulting questionnaires (eg, whether the inclusion of crowdsourcing-identified items would improve the internal consistency or responsiveness of questionnaires).

While our approach has revealed the relative importance of different LBP outcome indicators perceived by clinicians, the respondents’ rationales for choosing or prioritizing their answers remains unclear. Future qualitative research (eg, interviews or focus groups) should investigate clinician reasons for prioritizing various LBP outcome indicators and solicit information from older adults regarding their perceived important LBP outcome indicators. Collectively, our findings can be incorporated with patient and expert opinions obtained from qualitative and/or Delphi research to develop a new outcome measure for geriatric patients with LBP. This study has laid the foundation for developing better outcome measures for older patients with LBP. Such knowledge has the potential to ultimately contribute to better clinical management or treatment algorithms for older adults with LBP.

Overall, this is the first global crowdsourcing study to address LBP outcome questionnaire indicators for older adults. The study found that clinicians deemed functional improvements more important LBP outcome indicators for older adults with LBP than pain reduction or improvements of physical examinations. Clinicians generally perceive age-specific social functioning as an important outcome assessment domain for older adults with LBP. While further studies are warranted to compare our findings with the opinions obtained from older adults with LBP and/or leading spine experts, our study has laid the foundation for developing better outcome measures for older adults with LBP. In addition, this proof-of-concept study has also provided a framework to illustrate that global crowdsourcing approaches in spine research are viable and achievable, hopefully providing impetus for other investigators to adopt such an approach for future spine research.

## Appendix

Multimedia Appendix 1 List of professional organizations on Facebook and Twitter selected to advertise the project.

## Abbreviations

LBP: low back pain
