# Supplementary material for: Global Consensus From Clinicians Regarding Low Back Pain Outcome Indicators for Older Adults: Pairwise Wiki Survey Using Crowdsourcing
Source: JMIR Rehabil Assist Technol. 2019 Jan 15;6(1):e11127. doi: 10.2196/11127 (PMC6350088; doi:10.2196/11127)
Supplement: Multimedia Appendix 1 [file rehab_v6i1e11127_app1.pdf]

Multimedia Appendix 1. List of professional organizations on Facebook and Twitter selected to advertise the project.

**Target groups on Facebook**	
American Occupational Therapy Association	
American Physical Therapy Association	
Australian Association of Gerontology	
Australian & New Zealand Society for Geriatric Medicine	
Australian Medical Association	
Australian Physiotherapy Association	
British Columbia Chiropractic Association	
Canadian Chiropractic Association	
Evidence based chiropractic	
Gerontological Advanced Practice Nurses Association	
Gerontology institute of Georgia state university	
Global Health Division – Canadian Physiotherapy Association	
Hong Kong Chiropractors Association Ltd	
International osteopathy	
International osteopathic Association	
Japanese Association of Chiropractors	
McKenzie Institute Australia	
McKenzie Institute New Zealand	
McKenzie Institute USA	
McKenzie Mexico	
National Academy of Osteopathy	
North American Spine Society	
Occupational Therapy Australia	
Physiotherapy DPT Community	
Physiotherapy.Fisioterapia (European Region World Confederation for Physical Therapy)	
Physiotherapy Research Society - UK	
Royal Australian College of General Practitioners RACGP	
The American Geriatrics Society	
The Gerontological Society of America	
The Mckenzie Institute Japan	
The Physiotherapy Association of Trinidad and Tobago	
**Target groups on Twitter**	
Academy of Geriatric Physical Therapy	@AGPTtweets
American Chiropractic association	@ACAtoday
American Geriatrics Society	@AmericanGeriatricsSociety
American Physical Therapy Association	@APTAtweets
Australian Academy of Orthopaedic Manual Physical Therapists	@AAOMPT
Australian Association of Gerontology	@gerontologyau
Australian & New Zealand Society for Geriatric Medicine	@anzsgm
Australian Doctor	@australiandr
Australian physiotherapy association	@apaphysio
British Columbia Chiropractic Association	@bcchiro
British Geriatrics Society	@GeriSoc
Canadian Chiropractic Association	@canadianchiropracticassociation
Chartered Society of Physiotherapy	@CharteredPhysios @thecsp
European Region of World Confederation for Physical	@ERWCPT

Therapy	
Gerontological Advanced Practice Nurses Association	@GAPNA
Global Health Division – Canadian Physiotherapy Association	@IHDCanada
Gerontological Society of America	@geronsociety
International Association of Physical Therapists working with Older People	@iptopwcpt
International Federation of Orthopaedic Manipulative Physical Therapy	@IFOMPT
International osteopathic Association	@internationalosteopathicassociation
Irish Gerontology Society	#irishgerontsoc
Japanese Association of Chiropractors	@jac.chiropractic
JohnsHopkinsMedcine	@HopkinsMedicine
Mayo clinic	@Mayocliniic
McKenzie Institute New Zealand	@MDTNZ
McKenzie Institute USA	@McKenzieInstituteUSA
Mckenzie Institute Japan	@mdt.japan
McKenzie Mexico	@MDTMexico
Mulligan concept international	@MulliganConceptInternational
National Academy of Osteopathy	@osteopathycollege
North American Spine Society	@NASS.Spine
Occupational Therapy Australia	@otasut
Ontario Physiotherapy Association	@OntarioPT
Palmer College of Chiropractic	@palmercollege
Physiotherapy Association of British Columbia	@PhysiotherapyAssociationofBC
Physiotherapy Research Society – UK	@physioresearchsociety
Royal Australian College of General Practitioners	@RACGP
WCPT Physical Therapy Pain Network	@WCPTptpn
World Confederation for Physical Therapy	@WCPT
Young Geriatricians	@youngGeris
